# 2-[(3*S*,3a*S*,5*R*,8*S*,8a*S*)-3,8-Di­methyl­hexa­hydro-1*H*,4*H*-3a,8a-ep­oxy­azulen-5-yl]propan-2-ol

**DOI:** 10.1107/S1600536814013543

**Published:** 2014-06-14

**Authors:** Stacey Burrett, Dennis K. Taylor, Edward R. T. Tiekink

**Affiliations:** a School of Agriculture, Food and Wine, The University of Adelaide, Waite Campus, PMB 1, Glen Osmond, SA 5064, Australia; b Department of Chemistry, University of Malaya, 50603 Kuala Lumpur, Malaysia

## Abstract

Four independent mol­ecules (*A*–*D*) comprise the asymmetric unit of the title compound, C_15_H_26_O_2_, which differ only in the relative orientations of the terminal –C(Me)_2_OH groups [*e.g.* the range of C_methyl­ene_—C_methine_—C_quaternary_—O_hy­droxy_ torsion angles is 52.7 (7)–57.1 (6)°, where the C_methyl­ene_ atom is bound to an epoxide C atom]. The five-membered rings adopt envelope conformations, with the methyl­ene C atom adjacent to the methine C atom being the flap atom in each case. In each mol­ecule, the conformation of the seven-membered ring is a half-chair, with the C_methyl­ene_—C_methine_ bond, flanked by methyl­ene C atoms, being the back of the chair. Supra­molecular helical chains along the *b* axis are found in the crystal packing, sustained by hy­droxy–epoxide O—H⋯O hydrogen bonding. Mol­ecules of *A* self-associate into a chain as do those of *D*. A third independent chain comprising *B* and *C* mol­ecules is also formed. The studied crystal is a pseudo-merohedral twin (minor component *ca* 21%).

## Related literature   

For the preparation of the α- and β-epoxides of guaiol, see: Pesnelle (1966 ▶).
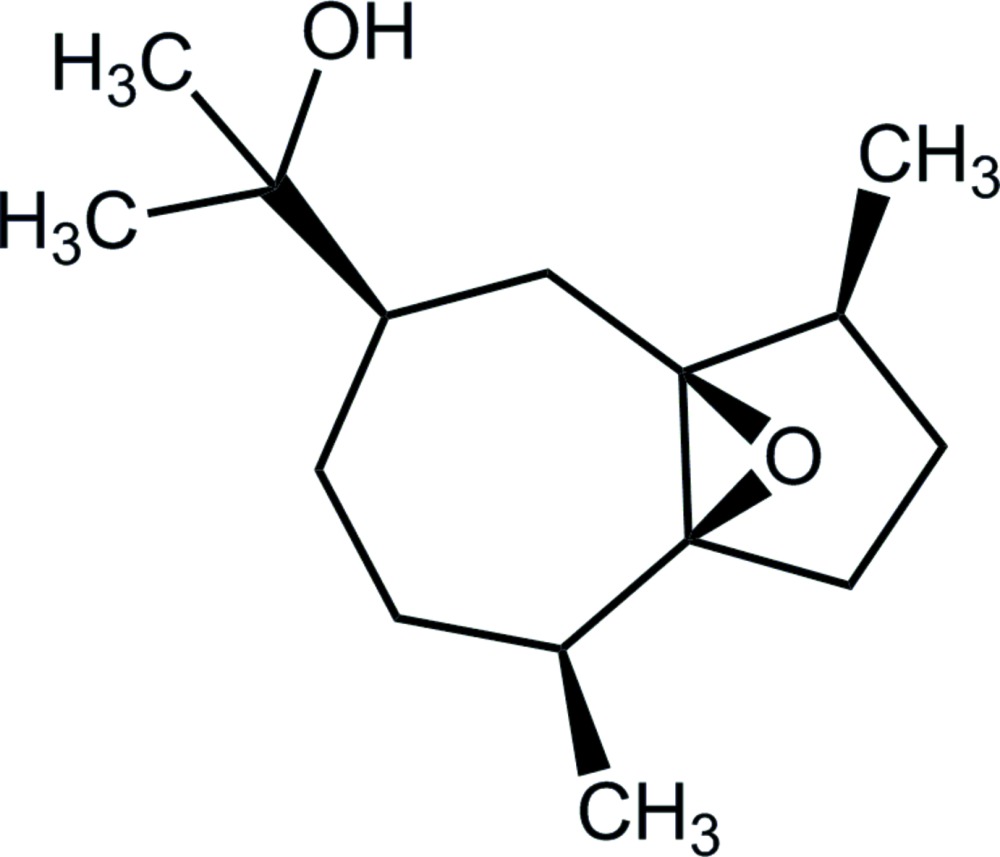


## Experimental   

### 

#### Crystal data  

C_15_H_26_O_2_
*M* *_r_* = 238.36
Monoclinic, 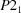

*a* = 7.4461 (1) Å
*b* = 11.0289 (2) Å
*c* = 33.7892 (6) Å
β = 90.579 (2)°
*V* = 2774.71 (8) Å^3^
*Z* = 8
Cu *K*α radiation
μ = 0.57 mm^−1^
*T* = 100 K
0.35 × 0.30 × 0.25 mm

#### Data collection  

Agilent SuperNova Dual diffractometer with an Atlas detector
Absorption correction: multi-scan (*CrysAlis PRO*; Agilent, 2013 ▶) *T*_min_ = 0.666, *T*_max_ = 1.000
21600 measured reflections
10038 independent reflections
9883 reflections with *I* > 2σ(*I*)
*R*_int_ = 0.050

#### Refinement  

*R*[*F* ^2^ > 2σ(*F* ^2^)] = 0.102
*wR*(*F* ^2^) = 0.278
*S* = 1.02
10038 reflections
618 parameters
1 restraint
H-atom parameters constrained
Δρ_max_ = 0.69 e Å^−3^
Δρ_min_ = −0.45 e Å^−3^

### 

Data collection: *CrysAlis PRO* (Agilent, 2013 ▶); cell refinement: *CrysAlis PRO*; data reduction: *CrysAlis PRO*; program(s) used to solve structure: *SHELXS97* (Sheldrick, 2008 ▶); program(s) used to refine structure: *SHELXL97* (Sheldrick, 2008 ▶); molecular graphics: *ORTEP-3 for Windows* (Farrugia, 2012 ▶) and *DIAMOND* (Brandenburg, 2006 ▶); software used to prepare material for publication: *publCIF* (Westrip, 2010 ▶).

## Supplementary Material

Crystal structure: contains datablock(s) general, I. DOI: 10.1107/S1600536814013543/su2742sup1.cif

Structure factors: contains datablock(s) I. DOI: 10.1107/S1600536814013543/su2742Isup2.hkl

CCDC reference: 1007725

Additional supporting information:  crystallographic information; 3D view; checkCIF report

## Figures and Tables

**Table 1:** Hydrogen-bond geometry (Å, °)

*D*—H⋯*A*	*D*—H	H⋯*A*	*D*⋯*A*	*D*—H⋯*A*
O2—H2⋯O1^i^	0.84	1.99	2.804 (6)	162
O4—H4⋯O5^ii^	0.84	2.00	2.792 (7)	157
O6—H6⋯O3	0.84	1.99	2.821 (7)	171
O8—H8⋯O7^iii^	0.84	2.00	2.795 (7)	158

Symmetry codes: (i) 
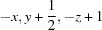
; (ii) 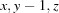; (iii) 
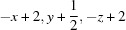
.

**Fig. 1.**
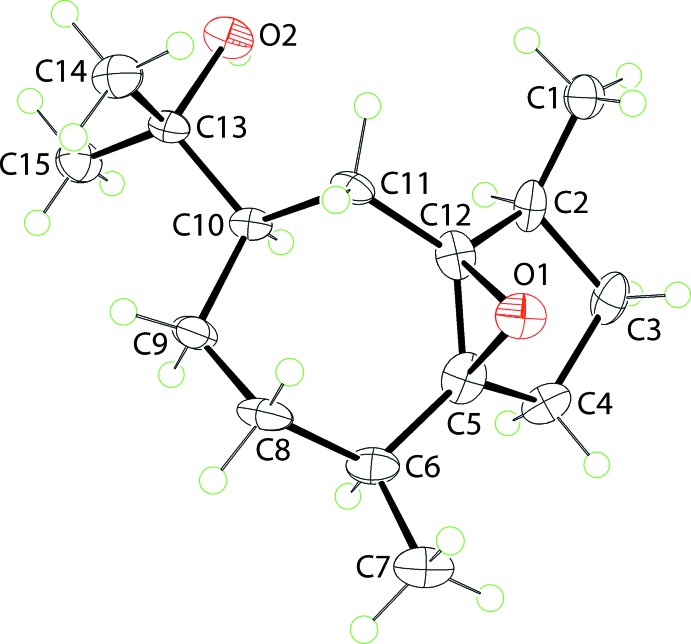
The molecular structure of the independent molecule A in (I), with atom labelling. The displacement ellipsoids are drawn at the 50% probability level. The other molecules are virtually identical and have sequential atom labelling.

**Fig. 2.**
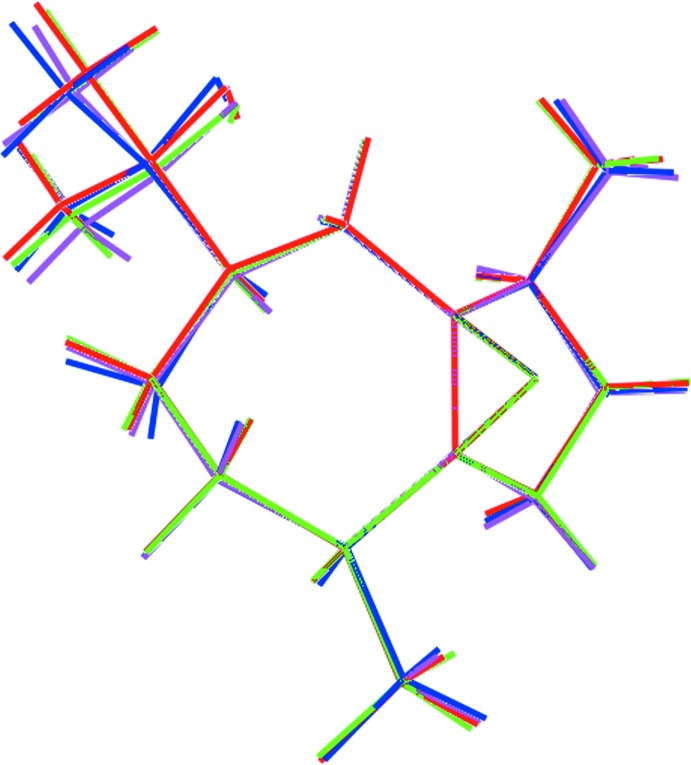
An overlay diagram of the four independent molecules (A-D) comprising the asymmetric unit in (I). The O1-, O3-, O5- and O7- containing molecules are coloured red, green, blue and pink, respectively. The molecules have been overlapped so that the epoxide rings are coincident.

**Fig. 3.**
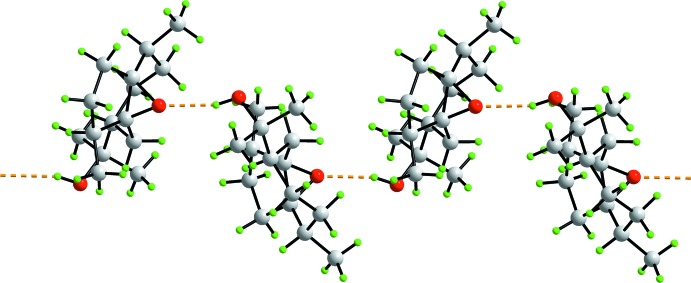
A view of the helical supramolecular chain along the b axis for the O1-containing molecule in (I). The O—H···O interactions are shown as orange dashed lines (see Table 1 for details).

## supplementary crystallographic information

### S2. Structural commentary

As has been reported previously (Pesnelle, 1966), the α and β-epoxides of guaiol have been prepared *via* the epoxidation of guaiol itself. In the present study suitable crystals for X-ray analysis of the minor product, β-ep­oxy guaiol (I), were isolated and the crystal structure is reported on herein.

Four independent molecules, *A*–*D*, comprise the crystallographic asymmetric unit of (I) with the O1-containing molecule shown in Fig. 1. As seen from the overlay diagram, Fig. 2, the four molecules are virtually superimposable with minor conformational differences noted in the relative orientations of the terminal -C(Me)_2_OH group as seen, for example, in the values of the C11—C10—C13—O2 (molecule *A*) and C56—C55—C58—O8 (*D*) torsion angles of 57.1 (6) and 52.7 (7)°, respectively. In each case, the five-membered ring has an envelope conformation with the C3, C18, C33 and C48 atoms, *i.e*. the atom adjacent to the methine-C atom, being the flap atom. The seven-membered rings are best described as being based on a half-chair conformation. In this description, for molecule *A*, the C9 and C10 atoms lie 1.051 (11) and 1.297 (9) Å, respectively, out of the mean plane defined by the remaining five atoms, *i.e*. C5, C6, C8, C11 and C12 (r.m.s. deviation = 0.0514 Å). The corresponding values for molecule *B* are 1.063 (10), 1.309 (8) (r.m.s. deviation = 0.0564 Å); for molecule *C* 1.144 (12), 1.293 (9) (r.m.s. deviation = 0.0303 Å); and for molecule *D* 1.067 (10), 1.309 (9) (r.m.s. deviation = 0.0526 Å). Finally, the oxygen atoms lie to opposite sides of the molecule.

The most prominent feature of the crystal packing is the formation of hydroxyl-O—H···O(epoxide) hydrogen bonding, Table 1, that leads to helical supra­molecular chains along the *b* axis. Molecules of *A* self-associate as illustrated in Fig. 3; molecules of *D* associate similarly. By contrast, molecules of *B* and *C* associate to form a third chain.

### S5. Synthesis and crystallization

A flask containing (-)-guaiol (4.01 g, 18 mmol) in di­chloro­methane (60 mL) was cooled to 273 K and peracetic acid in acetic acid (39%, 3.65 g, 18.7 mmol) was slowly added over 5 minutes. The mixture was stirred for an additional hour at 273 K and then washed with sodium bicarbonate (3 × 50 mL). The organic layer was dried over anhydrous MgSO_4_ and concentrated in vacuum and the residue purified by column chromatography (10:90, ether/hexane) to afford both the α and β-ep­oxy guaiols in a ratio of approximately 60:40. The minor β-isomer was recrystallised at 253 K from a small amount of ether hexane (10:90) to afford the title compound (1.43 g, 33%) as white crystals. M. pt: 323–326 K. Lit. (Pesnelle, 1966) M. pt: 317-319 K, R_f_ = 0.31 (50:50, EtOAc/hexane). Spectroscopic data for the title compound are available in the archived CIF.

### S6. Refinement

The H-atoms were placed in calculated positions and were included in the refinement in the riding model approximation: O—H = 0.84 Å, C—H = 0.95 - 1.00 Å with *U*_iso_(H) = 1.5*U*_eq_(O and C-methyl) and = 1.2U_eq_(C) for other H atoms. The studied crystal is a pseudo-merohedral twin [the fractional contribution of the minor component refined to 0.210 (3)], precluding the determination of the absolute structure. The latter was assigned based on the chemistry, i.e. the use of (-)-guaiol as reagent. The maximum and minimum residual electron density peaks of 0.69 and 0.45 eÅ^-3^, respectively, were located 1.02 Å and 0.76 Å from the O3 and C57 atoms, respectively.

### Crystal data

**Table:**

C_15_H_26_O_2_	*F*(000) = 1056
*M**_r_* = 238.36	*D*_x_ = 1.141 Mg m^−^^3^
Monoclinic, *P*2_1_	Cu *K*α radiation, λ = 1.54184 Å
Hall symbol: P 2yb	Cell parameters from 16057 reflections
*a* = 7.4461 (1) Å	θ = 2.2–74.2°
*b* = 11.0289 (2) Å	µ = 0.57 mm^−^^1^
*c* = 33.7892 (6) Å	*T* = 100 K
β = 90.579 (2)°	Block, colourless
*V* = 2774.71 (8) Å^3^	0.35 × 0.30 × 0.25 mm
*Z* = 8	

### Data collection

**Table:**

Agilent SuperNova Dual diffractometer with an Atlas detector	10038 independent reflections
Radiation source: SuperNova (Cu) X-ray Source	9883 reflections with *I* > 2σ(*I*)
Mirror monochromator	*R*_int_ = 0.050
Detector resolution: 10.4041 pixels mm^-1^	θ_max_ = 74.4°, θ_min_ = 2.6°
ω scan	*h* = −8→6
Absorption correction: multi-scan (*CrysAlis PRO*; Agilent, 2013)	*k* = −13→13
*T*_min_ = 0.666, *T*_max_ = 1.000	*l* = −42→41
21600 measured reflections	

### Refinement

**Table:**

Refinement on *F*^2^	Primary atom site location: structure-invariant direct methods
Least-squares matrix: full	Secondary atom site location: difference Fourier map
*R*[*F*^2^ > 2σ(*F*^2^)] = 0.102	Hydrogen site location: inferred from neighbouring sites
*wR*(*F*^2^) = 0.278	H-atom parameters constrained
*S* = 1.02	*w* = 1/[σ^2^(*F*_o_^2^) + (0.1597*P*)^2^ + 8.6322*P*] where *P* = (*F*_o_^2^ + 2*F*_c_^2^)/3
10038 reflections	(Δ/σ)_max_ = 0.001
618 parameters	Δρ_max_ = 0.69 e Å^−^^3^
1 restraint	Δρ_min_ = −0.45 e Å^−^^3^

### Special details

**Table:**

Experimental. Specroscopic data for the title compound: ^1^H NMR (500 MHz, CDCl_3_) δ 2.08 (d, J = 15.0 Hz, 1H), 1.98 (dd, J = 10.0, 5.0 Hz, 1H), 1.98-1.89 (m, 2H), 1.70-1.63 (m, 1H), 1.62-1.44 (m, 4H), 1.37-1.31 (m, 2H), 1.27-1.23 (m, 1H), 1.17 (s, 3H), 1.13 (s, 3H), 1.04 (d, J= 7.2 Hz, 3H), 1.03 (d, J = 7.2, 3H), 0.98-0.92 (m, 1H). ^13^C NMR (300 MHz, CDCl_3_) δ 74.3, 72.9, 72.1, 45.8, 37.0, 34.1, 30.6, 27.9, 27.4, 27.3, 27.2, 25.8, 24.8, 18.4, 13.3. MS m/z 238 (1.0), 220 (12), 205 (14), 187 (9), 177 (8), 165 (36), 156 (22), 147 (25), 138 (29), 125 (43), 123 (51), 109 (36), 95 (43), 81 (38), 67 (38), 59 (100). All other physical and spectral data were identical to those previously reported by Pesnelle (1966).

### Fractional atomic coordinates and isotropic or equivalent isotropic displacement parameters (Å^2^)

**Table:**

	*x*	*y*	*z*	*U*_iso_*/*U*_eq_	
O1	0.1637 (6)	0.0019 (4)	0.55216 (14)	0.0264 (9)	
O2	−0.2032 (6)	0.2494 (4)	0.44318 (14)	0.0263 (10)	
H2	−0.1686	0.3214	0.4461	0.039*	
O3	0.3719 (6)	0.1231 (4)	0.80228 (12)	0.0235 (9)	
O4	0.7098 (6)	−0.1582 (4)	0.70230 (15)	0.0308 (11)	
H4	0.6926	−0.2330	0.6995	0.046*	
O5	0.7594 (6)	0.5923 (4)	0.69356 (13)	0.0244 (9)	
O6	0.3269 (6)	0.3766 (4)	0.79755 (15)	0.0270 (10)	
H6	0.3530	0.3028	0.7995	0.040*	
O7	0.7675 (6)	0.5159 (4)	0.94286 (12)	0.0220 (9)	
O8	1.1765 (8)	0.7721 (5)	1.03806 (14)	0.0347 (12)	
H8	1.1631	0.8457	1.0437	0.052*	
C1	−0.1515 (9)	0.1911 (7)	0.57239 (19)	0.0291 (14)	
H1A	−0.2372	0.1885	0.5502	0.044*	
H1B	−0.1812	0.2589	0.5899	0.044*	
H1C	−0.1578	0.1149	0.5872	0.044*	
C2	0.0361 (10)	0.2078 (6)	0.55675 (17)	0.0265 (14)	
H2A	0.0385	0.2852	0.5414	0.032*	
C3	0.1834 (10)	0.2147 (6)	0.58879 (18)	0.0304 (15)	
H3A	0.1952	0.2983	0.5991	0.036*	
H3B	0.1560	0.1596	0.6111	0.036*	
C4	0.3554 (9)	0.1752 (6)	0.5680 (2)	0.0279 (13)	
H4A	0.4168	0.2458	0.5561	0.033*	
H4B	0.4389	0.1351	0.5868	0.033*	
C5	0.2935 (10)	0.0868 (5)	0.53601 (19)	0.0259 (13)	
C6	0.4287 (8)	0.0396 (7)	0.5062 (2)	0.0276 (14)	
H6A	0.5077	0.1089	0.4985	0.033*	
C7	0.5472 (9)	−0.0569 (7)	0.5254 (2)	0.0329 (15)	
H7A	0.6040	−0.0237	0.5494	0.049*	
H7B	0.6402	−0.0820	0.5068	0.049*	
H7C	0.4736	−0.1272	0.5325	0.049*	
C8	0.3397 (9)	−0.0099 (7)	0.4681 (2)	0.0291 (14)	
H8A	0.4334	−0.0516	0.4526	0.035*	
H8B	0.2509	−0.0721	0.4759	0.035*	
C9	0.2454 (8)	0.0797 (7)	0.44084 (19)	0.0280 (14)	
H9A	0.2058	0.0352	0.4168	0.034*	
H9B	0.3351	0.1404	0.4324	0.034*	
C10	0.0818 (8)	0.1488 (5)	0.45703 (17)	0.0189 (11)	
H10	0.1275	0.2279	0.4675	0.023*	
C11	−0.0019 (8)	0.0808 (6)	0.49218 (17)	0.0200 (11)	
H11A	−0.1284	0.1065	0.4952	0.024*	
H11B	−0.0007	−0.0074	0.4869	0.024*	
C12	0.1011 (9)	0.1067 (5)	0.52990 (18)	0.0233 (13)	
C13	−0.0622 (8)	0.1791 (6)	0.42525 (18)	0.0219 (12)	
C14	−0.1557 (9)	0.0671 (6)	0.4092 (2)	0.0296 (14)	
H14A	−0.2449	0.0911	0.3892	0.044*	
H14B	−0.2156	0.0246	0.4308	0.044*	
H14C	−0.0669	0.0133	0.3971	0.044*	
C15	0.0192 (10)	0.2517 (7)	0.3914 (2)	0.0310 (14)	
H15A	−0.0742	0.2696	0.3715	0.046*	
H15B	0.1150	0.2043	0.3791	0.046*	
H15C	0.0689	0.3278	0.4017	0.046*	
C16	0.6953 (9)	−0.0599 (7)	0.82496 (18)	0.0275 (13)	
H16A	0.7770	−0.0627	0.8024	0.041*	
H16B	0.7295	−0.1226	0.8442	0.041*	
H16C	0.7028	0.0200	0.8376	0.041*	
C17	0.5062 (9)	−0.0819 (5)	0.81076 (17)	0.0225 (12)	
H17	0.5019	−0.1626	0.7973	0.027*	
C18	0.3648 (9)	−0.0814 (6)	0.84396 (18)	0.0254 (13)	
H18A	0.3972	−0.0218	0.8648	0.030*	
H18B	0.3545	−0.1626	0.8562	0.030*	
C19	0.1871 (8)	−0.0451 (6)	0.82303 (17)	0.0227 (12)	
H19A	0.1212	−0.1179	0.8138	0.027*	
H19B	0.1092	0.0011	0.8412	0.027*	
C20	0.2424 (9)	0.0325 (5)	0.78846 (17)	0.0220 (12)	
C21	0.1027 (8)	0.0731 (5)	0.75806 (17)	0.0201 (12)	
H21	0.0238	0.0019	0.7521	0.024*	
C22	−0.0153 (8)	0.1720 (6)	0.7756 (2)	0.0247 (13)	
H22A	−0.1043	0.1981	0.7558	0.037*	
H22B	0.0596	0.2412	0.7834	0.037*	
H22C	−0.0771	0.1402	0.7989	0.037*	
C23	0.1825 (9)	0.1157 (6)	0.71879 (17)	0.0258 (13)	
H23A	0.2711	0.1803	0.7247	0.031*	
H23B	0.0847	0.1530	0.7029	0.031*	
C24	0.2758 (9)	0.0190 (6)	0.69273 (17)	0.0247 (13)	
H24A	0.1852	−0.0427	0.6852	0.030*	
H24B	0.3153	0.0590	0.6681	0.030*	
C25	0.4376 (7)	−0.0466 (5)	0.71093 (16)	0.0168 (11)	
H25	0.3891	−0.1201	0.7244	0.020*	
C26	0.5282 (8)	0.0300 (5)	0.74327 (16)	0.0185 (11)	
H26A	0.6556	0.0056	0.7461	0.022*	
H26B	0.5246	0.1166	0.7356	0.022*	
C27	0.4349 (8)	0.0134 (5)	0.78202 (17)	0.0193 (11)	
C28	0.5770 (8)	−0.0931 (6)	0.68052 (18)	0.0231 (13)	
C29	0.6720 (11)	0.0105 (7)	0.66018 (18)	0.0324 (15)	
H29A	0.7585	−0.0220	0.6413	0.049*	
H29B	0.7355	0.0598	0.6800	0.049*	
H29C	0.5837	0.0608	0.6461	0.049*	
C30	0.4856 (10)	−0.1748 (8)	0.6498 (2)	0.0350 (16)	
H30A	0.5748	−0.2032	0.6308	0.052*	
H30B	0.3917	−0.1290	0.6358	0.052*	
H30C	0.4317	−0.2447	0.6631	0.052*	
C31	0.4955 (11)	0.3829 (7)	0.6660 (2)	0.0336 (15)	
H31A	0.3882	0.3908	0.6823	0.050*	
H31B	0.4879	0.3079	0.6505	0.050*	
H31C	0.5035	0.4525	0.6480	0.050*	
C32	0.6622 (9)	0.3790 (6)	0.69258 (18)	0.0235 (12)	
H32	0.6532	0.3073	0.7106	0.028*	
C33	0.8374 (9)	0.3682 (6)	0.66847 (16)	0.0240 (13)	
H33A	0.8678	0.2820	0.6638	0.029*	
H33B	0.8240	0.4094	0.6426	0.029*	
C34	0.9828 (9)	0.4295 (6)	0.69350 (17)	0.0236 (12)	
H34A	1.0460	0.3697	0.7105	0.028*	
H34B	1.0717	0.4706	0.6765	0.028*	
C35	0.8810 (9)	0.5208 (5)	0.71837 (17)	0.0236 (13)	
C36	0.9850 (8)	0.5873 (6)	0.75028 (18)	0.0226 (12)	
H36	1.0695	0.5271	0.7624	0.027*	
C37	1.1002 (10)	0.6864 (6)	0.7322 (2)	0.0310 (15)	
H37A	1.1734	0.6519	0.7111	0.046*	
H37B	1.1789	0.7211	0.7527	0.046*	
H37C	1.0225	0.7500	0.7213	0.046*	
C38	0.8717 (9)	0.6376 (6)	0.78383 (19)	0.0267 (14)	
H38A	0.7800	0.6927	0.7724	0.032*	
H38B	0.9502	0.6866	0.8014	0.032*	
C39	0.7762 (9)	0.5417 (8)	0.8090 (2)	0.0359 (17)	
H39A	0.7272	0.5830	0.8325	0.043*	
H39B	0.8680	0.4835	0.8185	0.043*	
C40	0.6210 (8)	0.4676 (6)	0.78949 (17)	0.0225 (12)	
H40	0.6710	0.3855	0.7835	0.027*	
C41	0.5595 (8)	0.5223 (6)	0.74944 (18)	0.0249 (13)	
H41A	0.4398	0.4897	0.7421	0.030*	
H41B	0.5492	0.6114	0.7519	0.030*	
C42	0.6929 (9)	0.4916 (5)	0.71739 (17)	0.0222 (12)	
C43	0.4597 (8)	0.4474 (6)	0.81784 (19)	0.0229 (12)	
C44	0.5221 (10)	0.3784 (7)	0.8548 (2)	0.0311 (14)	
H44A	0.4200	0.3661	0.8724	0.047*	
H44B	0.6153	0.4253	0.8686	0.047*	
H44C	0.5710	0.2995	0.8470	0.047*	
C45	0.3653 (10)	0.5643 (6)	0.8287 (2)	0.0292 (14)	
H45A	0.2656	0.5464	0.8465	0.044*	
H45B	0.3187	0.6032	0.8046	0.044*	
H45C	0.4505	0.6188	0.8420	0.044*	
C46	1.0319 (10)	0.7138 (7)	0.9105 (2)	0.0340 (16)	
H46A	1.1418	0.7046	0.9264	0.051*	
H46B	1.0417	0.7861	0.8938	0.051*	
H46C	1.0155	0.6421	0.8937	0.051*	
C47	0.8719 (9)	0.7270 (6)	0.93767 (16)	0.0238 (13)	
H47	0.8893	0.8009	0.9544	0.029*	
C48	0.6900 (8)	0.7390 (6)	0.91433 (17)	0.0235 (13)	
H48A	0.6604	0.8254	0.9099	0.028*	
H48B	0.6986	0.6984	0.8883	0.028*	
C49	0.5451 (9)	0.6782 (6)	0.93949 (18)	0.0241 (13)	
H49A	0.4796	0.7389	0.9554	0.029*	
H49B	0.4580	0.6336	0.9226	0.029*	
C50	0.6499 (10)	0.5920 (6)	0.96589 (16)	0.0243 (13)	
C51	0.5465 (8)	0.5341 (6)	0.99949 (18)	0.0209 (12)	
H51	0.4682	0.5986	1.0109	0.025*	
C52	0.4218 (9)	0.4353 (7)	0.98339 (19)	0.0288 (14)	
H52A	0.3474	0.4686	0.9619	0.043*	
H52B	0.3445	0.4060	1.0046	0.043*	
H52C	0.4938	0.3678	0.9733	0.043*	
C53	0.6616 (8)	0.4845 (6)	1.03340 (17)	0.0234 (13)	
H53A	0.7482	0.4258	1.0223	0.028*	
H53B	0.5826	0.4391	1.0515	0.028*	
C54	0.7671 (10)	0.5783 (7)	1.05779 (18)	0.0291 (14)	
H54A	0.8314	0.5347	1.0793	0.035*	
H54B	0.6794	0.6330	1.0705	0.035*	
C55	0.9050 (8)	0.6576 (6)	1.03592 (16)	0.0202 (12)	
H55	0.8399	0.7326	1.0274	0.024*	
C56	0.9721 (8)	0.5963 (6)	0.99793 (16)	0.0206 (12)	
H56A	1.0904	0.6301	0.9907	0.025*	
H56B	0.9863	0.5081	1.0024	0.025*	
C57	0.8369 (8)	0.6185 (5)	0.96457 (16)	0.0179 (11)	
C58	1.0610 (10)	0.6993 (6)	1.06280 (17)	0.0256 (14)	
C59	0.9908 (12)	0.7763 (7)	1.09692 (19)	0.0370 (18)	
H59A	1.0916	0.8019	1.1138	0.055*	
H59B	0.9063	0.7284	1.1126	0.055*	
H59C	0.9294	0.8480	1.0863	0.055*	
C60	1.1761 (10)	0.5952 (7)	1.07805 (18)	0.0312 (15)	
H60A	1.2201	0.5477	1.0556	0.047*	
H60B	1.1041	0.5431	1.0952	0.047*	
H60C	1.2784	0.6276	1.0931	0.047*	

### Atomic displacement parameters (Å^2^)

**Table:**

	*U*^11^	*U*^22^	*U*^33^	*U*^12^	*U*^13^	*U*^23^
O1	0.029 (2)	0.014 (2)	0.036 (2)	−0.0009 (18)	0.0010 (18)	0.0051 (18)
O2	0.028 (2)	0.012 (2)	0.040 (2)	0.0034 (17)	0.0041 (19)	−0.0029 (18)
O3	0.028 (2)	0.018 (2)	0.024 (2)	−0.0006 (18)	−0.0006 (17)	−0.0035 (17)
O4	0.027 (2)	0.021 (2)	0.044 (3)	0.0116 (19)	−0.008 (2)	−0.006 (2)
O5	0.029 (2)	0.016 (2)	0.028 (2)	−0.0012 (18)	−0.0015 (17)	0.0042 (17)
O6	0.024 (2)	0.013 (2)	0.043 (2)	−0.0068 (18)	−0.0053 (19)	−0.0004 (19)
O7	0.026 (2)	0.015 (2)	0.026 (2)	−0.0014 (17)	0.0038 (16)	−0.0038 (17)
O8	0.054 (3)	0.023 (2)	0.027 (2)	−0.018 (2)	0.011 (2)	−0.0036 (19)
C1	0.030 (4)	0.033 (4)	0.024 (3)	0.000 (3)	0.000 (2)	−0.005 (3)
C2	0.043 (4)	0.020 (3)	0.016 (3)	0.002 (3)	−0.001 (2)	0.000 (2)
C3	0.048 (4)	0.022 (3)	0.021 (3)	0.005 (3)	−0.008 (3)	−0.006 (2)
C4	0.028 (3)	0.020 (3)	0.036 (3)	−0.001 (3)	−0.009 (3)	−0.001 (3)
C5	0.041 (4)	0.007 (3)	0.030 (3)	−0.001 (3)	−0.003 (3)	0.003 (2)
C6	0.016 (3)	0.026 (3)	0.041 (4)	−0.006 (3)	−0.001 (2)	−0.002 (3)
C7	0.022 (3)	0.027 (4)	0.050 (4)	0.001 (3)	0.000 (3)	0.002 (3)
C8	0.014 (3)	0.028 (3)	0.045 (4)	0.003 (2)	0.003 (3)	−0.011 (3)
C9	0.016 (3)	0.036 (4)	0.032 (3)	0.003 (3)	0.004 (2)	−0.007 (3)
C10	0.015 (3)	0.013 (3)	0.028 (3)	−0.001 (2)	0.001 (2)	−0.002 (2)
C11	0.014 (3)	0.018 (3)	0.028 (3)	−0.004 (2)	0.004 (2)	−0.007 (2)
C12	0.034 (3)	0.012 (3)	0.024 (3)	−0.007 (2)	0.004 (2)	−0.001 (2)
C13	0.015 (3)	0.024 (3)	0.027 (3)	−0.001 (2)	0.002 (2)	0.000 (2)
C14	0.027 (3)	0.024 (3)	0.038 (3)	0.000 (3)	−0.003 (3)	−0.009 (3)
C15	0.029 (3)	0.031 (4)	0.033 (3)	0.001 (3)	0.003 (3)	0.006 (3)
C16	0.032 (3)	0.027 (3)	0.024 (3)	0.002 (3)	0.003 (2)	0.004 (2)
C17	0.034 (3)	0.012 (3)	0.021 (3)	−0.002 (2)	0.005 (2)	−0.001 (2)
C18	0.033 (3)	0.021 (3)	0.022 (3)	0.006 (3)	−0.001 (2)	0.002 (2)
C19	0.023 (3)	0.019 (3)	0.026 (3)	−0.004 (2)	0.004 (2)	0.002 (2)
C20	0.038 (3)	0.007 (3)	0.021 (3)	−0.001 (2)	−0.003 (2)	−0.001 (2)
C21	0.019 (3)	0.014 (3)	0.028 (3)	0.001 (2)	−0.002 (2)	−0.002 (2)
C22	0.009 (3)	0.024 (3)	0.041 (3)	0.002 (2)	0.005 (2)	0.001 (3)
C23	0.029 (3)	0.025 (3)	0.024 (3)	0.012 (3)	−0.002 (2)	0.000 (2)
C24	0.031 (3)	0.021 (3)	0.022 (3)	0.008 (3)	−0.004 (2)	0.004 (2)
C25	0.012 (2)	0.013 (3)	0.026 (3)	−0.002 (2)	0.001 (2)	0.002 (2)
C26	0.022 (3)	0.012 (3)	0.021 (3)	−0.002 (2)	0.000 (2)	0.003 (2)
C27	0.025 (3)	0.009 (3)	0.024 (3)	−0.004 (2)	−0.004 (2)	−0.001 (2)
C28	0.017 (3)	0.023 (3)	0.029 (3)	0.004 (2)	0.001 (2)	−0.004 (2)
C29	0.050 (4)	0.027 (4)	0.020 (3)	−0.001 (3)	0.008 (3)	0.002 (2)
C30	0.034 (4)	0.041 (4)	0.031 (3)	0.002 (3)	−0.003 (3)	−0.012 (3)
C31	0.042 (4)	0.030 (4)	0.029 (3)	0.002 (3)	−0.005 (3)	−0.008 (3)
C32	0.028 (3)	0.017 (3)	0.026 (3)	−0.001 (3)	−0.002 (2)	0.000 (2)
C33	0.040 (4)	0.013 (3)	0.019 (3)	0.001 (3)	0.005 (2)	−0.002 (2)
C34	0.034 (3)	0.013 (3)	0.023 (3)	0.005 (2)	−0.001 (2)	0.001 (2)
C35	0.041 (4)	0.008 (3)	0.022 (3)	0.003 (2)	−0.003 (2)	0.005 (2)
C36	0.023 (3)	0.015 (3)	0.030 (3)	−0.003 (2)	0.002 (2)	−0.002 (2)
C37	0.037 (4)	0.016 (3)	0.039 (3)	−0.005 (3)	−0.001 (3)	0.004 (3)
C38	0.022 (3)	0.022 (3)	0.036 (3)	−0.006 (2)	0.003 (2)	−0.016 (3)
C39	0.025 (3)	0.055 (5)	0.028 (3)	−0.014 (3)	0.000 (2)	−0.012 (3)
C40	0.018 (3)	0.025 (3)	0.025 (3)	0.003 (2)	0.001 (2)	−0.007 (2)
C41	0.019 (3)	0.029 (3)	0.026 (3)	0.002 (3)	−0.001 (2)	−0.007 (3)
C42	0.035 (3)	0.009 (3)	0.022 (3)	0.002 (2)	0.003 (2)	0.001 (2)
C43	0.021 (3)	0.015 (3)	0.033 (3)	−0.006 (2)	−0.004 (2)	−0.001 (2)
C44	0.034 (4)	0.022 (3)	0.038 (3)	0.004 (3)	0.000 (3)	−0.001 (3)
C45	0.031 (3)	0.022 (3)	0.034 (3)	−0.004 (3)	0.003 (3)	−0.005 (3)
C46	0.035 (4)	0.039 (4)	0.027 (3)	0.005 (3)	0.008 (3)	0.007 (3)
C47	0.040 (4)	0.017 (3)	0.014 (2)	0.001 (3)	−0.003 (2)	0.000 (2)
C48	0.027 (3)	0.024 (3)	0.019 (3)	0.003 (3)	0.000 (2)	0.003 (2)
C49	0.024 (3)	0.022 (3)	0.026 (3)	−0.001 (3)	0.001 (2)	−0.002 (2)
C50	0.044 (4)	0.012 (3)	0.017 (2)	0.009 (3)	0.000 (2)	−0.005 (2)
C51	0.019 (3)	0.014 (3)	0.030 (3)	0.000 (2)	0.002 (2)	−0.001 (2)
C52	0.029 (3)	0.028 (4)	0.030 (3)	−0.001 (3)	0.001 (2)	−0.007 (3)
C53	0.021 (3)	0.026 (3)	0.023 (3)	−0.010 (2)	0.004 (2)	0.002 (2)
C54	0.038 (4)	0.028 (3)	0.021 (3)	−0.010 (3)	0.004 (2)	−0.002 (3)
C55	0.023 (3)	0.016 (3)	0.021 (3)	−0.003 (2)	−0.002 (2)	0.004 (2)
C56	0.024 (3)	0.022 (3)	0.016 (2)	0.006 (2)	−0.001 (2)	0.003 (2)
C57	0.026 (3)	0.008 (2)	0.019 (2)	0.002 (2)	0.001 (2)	−0.002 (2)
C58	0.038 (4)	0.019 (3)	0.020 (3)	−0.015 (3)	0.002 (2)	0.002 (2)
C59	0.057 (5)	0.031 (4)	0.024 (3)	−0.021 (3)	0.004 (3)	−0.008 (3)
C60	0.032 (3)	0.038 (4)	0.024 (3)	−0.002 (3)	−0.003 (2)	0.006 (3)

### Geometric parameters (Å, º)

**Table:**

O1—C12	1.454 (8)	C28—C29	1.513 (9)
O1—C5	1.456 (8)	C28—C30	1.529 (9)
O2—C13	1.443 (7)	C29—H29A	0.9800
O2—H2	0.8400	C29—H29B	0.9800
O3—C20	1.462 (7)	C29—H29C	0.9800
O3—C27	1.469 (7)	C30—H30A	0.9800
O4—C28	1.421 (7)	C30—H30B	0.9800
O4—H4	0.8400	C30—H30C	0.9800
O5—C35	1.460 (7)	C31—C32	1.526 (9)
O5—C42	1.462 (7)	C31—H31A	0.9800
O6—C43	1.429 (7)	C31—H31B	0.9800
O6—H6	0.8400	C31—H31C	0.9800
O7—C57	1.442 (7)	C32—C42	1.514 (8)
O7—C50	1.446 (7)	C32—C33	1.550 (9)
O8—C58	1.448 (7)	C32—H32	1.0000
O8—H8	0.8400	C33—C34	1.525 (9)
C1—C2	1.509 (10)	C33—H33A	0.9900
C1—H1A	0.9800	C33—H33B	0.9900
C1—H1B	0.9800	C34—C35	1.519 (8)
C1—H1C	0.9800	C34—H34A	0.9900
C2—C12	1.520 (9)	C34—H34B	0.9900
C2—C3	1.535 (9)	C35—C42	1.437 (10)
C2—H2A	1.0000	C35—C36	1.511 (8)
C3—C4	1.531 (10)	C36—C37	1.521 (9)
C3—H3A	0.9900	C36—C38	1.525 (8)
C3—H3B	0.9900	C36—H36	1.0000
C4—C5	1.523 (9)	C37—H37A	0.9800
C4—H4A	0.9900	C37—H37B	0.9800
C4—H4B	0.9900	C37—H37C	0.9800
C5—C12	1.462 (10)	C38—C39	1.536 (10)
C5—C6	1.523 (9)	C38—H38A	0.9900
C6—C7	1.525 (10)	C38—H38B	0.9900
C6—C8	1.542 (9)	C39—C40	1.556 (9)
C6—H6A	1.0000	C39—H39A	0.9900
C7—H7A	0.9800	C39—H39B	0.9900
C7—H7B	0.9800	C40—C41	1.547 (8)
C7—H7C	0.9800	C40—C43	1.560 (8)
C8—C9	1.518 (10)	C40—H40	1.0000
C8—H8A	0.9900	C41—C42	1.515 (8)
C8—H8B	0.9900	C41—H41A	0.9900
C9—C10	1.542 (8)	C41—H41B	0.9900
C9—H9A	0.9900	C43—C45	1.515 (9)
C9—H9B	0.9900	C43—C44	1.530 (9)
C10—C11	1.541 (8)	C44—H44A	0.9800
C10—C13	1.546 (8)	C44—H44B	0.9800
C10—H10	1.0000	C44—H44C	0.9800
C11—C12	1.508 (8)	C45—H45A	0.9800
C11—H11A	0.9900	C45—H45B	0.9800
C11—H11B	0.9900	C45—H45C	0.9800
C13—C14	1.515 (9)	C46—C47	1.519 (9)
C13—C15	1.528 (9)	C46—H46A	0.9800
C14—H14A	0.9800	C46—H46B	0.9800
C14—H14B	0.9800	C46—H46C	0.9800
C14—H14C	0.9800	C47—C57	1.527 (8)
C15—H15A	0.9800	C47—C48	1.566 (9)
C15—H15B	0.9800	C47—H47	1.0000
C15—H15C	0.9800	C48—C49	1.535 (9)
C16—C17	1.503 (10)	C48—H48A	0.9900
C16—H16A	0.9800	C48—H48B	0.9900
C16—H16B	0.9800	C49—C50	1.515 (9)
C16—H16C	0.9800	C49—H49A	0.9900
C17—C27	1.523 (8)	C49—H49B	0.9900
C17—C18	1.546 (8)	C50—C57	1.424 (10)
C17—H17	1.0000	C50—C51	1.519 (9)
C18—C19	1.547 (9)	C51—C53	1.526 (8)
C18—H18A	0.9900	C51—C52	1.528 (9)
C18—H18B	0.9900	C51—H51	1.0000
C19—C20	1.509 (8)	C52—H52A	0.9800
C19—H19A	0.9900	C52—H52B	0.9800
C19—H19B	0.9900	C52—H52C	0.9800
C20—C27	1.467 (9)	C53—C54	1.534 (9)
C20—C21	1.522 (8)	C53—H53A	0.9900
C21—C22	1.525 (8)	C53—H53B	0.9900
C21—C23	1.533 (8)	C54—C55	1.543 (8)
C21—H21	1.0000	C54—H54A	0.9900
C22—H22A	0.9800	C54—H54B	0.9900
C22—H22B	0.9800	C55—C58	1.538 (8)
C22—H22C	0.9800	C55—C56	1.538 (8)
C23—C24	1.552 (9)	C55—H55	1.0000
C23—H23A	0.9900	C56—C57	1.523 (8)
C23—H23B	0.9900	C56—H56A	0.9900
C24—C25	1.529 (8)	C56—H56B	0.9900
C24—H24A	0.9900	C58—C60	1.520 (10)
C24—H24B	0.9900	C58—C59	1.529 (9)
C25—C26	1.532 (8)	C59—H59A	0.9800
C25—C28	1.555 (8)	C59—H59B	0.9800
C25—H25	1.0000	C59—H59C	0.9800
C26—C27	1.500 (8)	C60—H60A	0.9800
C26—H26A	0.9900	C60—H60B	0.9800
C26—H26B	0.9900	C60—H60C	0.9800
			
C12—O1—C5	60.3 (4)	H30A—C30—H30B	109.5
C13—O2—H2	109.5	C28—C30—H30C	109.5
C20—O3—C27	60.1 (4)	H30A—C30—H30C	109.5
C28—O4—H4	109.5	H30B—C30—H30C	109.5
C35—O5—C42	58.9 (4)	C32—C31—H31A	109.5
C43—O6—H6	109.5	C32—C31—H31B	109.5
C57—O7—C50	59.1 (4)	H31A—C31—H31B	109.5
C58—O8—H8	109.5	C32—C31—H31C	109.5
C2—C1—H1A	109.5	H31A—C31—H31C	109.5
C2—C1—H1B	109.5	H31B—C31—H31C	109.5
H1A—C1—H1B	109.5	C42—C32—C31	114.8 (6)
C2—C1—H1C	109.5	C42—C32—C33	103.3 (5)
H1A—C1—H1C	109.5	C31—C32—C33	112.1 (5)
H1B—C1—H1C	109.5	C42—C32—H32	108.8
C1—C2—C12	114.9 (6)	C31—C32—H32	108.8
C1—C2—C3	114.6 (5)	C33—C32—H32	108.8
C12—C2—C3	103.2 (6)	C34—C33—C32	105.7 (5)
C1—C2—H2A	107.9	C34—C33—H33A	110.6
C12—C2—H2A	107.9	C32—C33—H33A	110.6
C3—C2—H2A	107.9	C34—C33—H33B	110.6
C4—C3—C2	104.9 (5)	C32—C33—H33B	110.6
C4—C3—H3A	110.8	H33A—C33—H33B	108.7
C2—C3—H3A	110.8	C35—C34—C33	104.2 (5)
C4—C3—H3B	110.8	C35—C34—H34A	110.9
C2—C3—H3B	110.8	C33—C34—H34A	110.9
H3A—C3—H3B	108.8	C35—C34—H34B	110.9
C5—C4—C3	105.0 (5)	C33—C34—H34B	110.9
C5—C4—H4A	110.7	H34A—C34—H34B	108.9
C3—C4—H4A	110.7	C42—C35—O5	60.6 (4)
C5—C4—H4B	110.7	C42—C35—C36	128.1 (6)
C3—C4—H4B	110.7	O5—C35—C36	117.2 (5)
H4A—C4—H4B	108.8	C42—C35—C34	109.3 (5)
O1—C5—C12	59.7 (4)	O5—C35—C34	110.5 (5)
O1—C5—C4	110.1 (5)	C36—C35—C34	117.5 (6)
C12—C5—C4	107.1 (6)	C35—C36—C37	110.5 (5)
O1—C5—C6	118.3 (5)	C35—C36—C38	115.1 (5)
C12—C5—C6	127.6 (6)	C37—C36—C38	110.8 (5)
C4—C5—C6	119.4 (6)	C35—C36—H36	106.7
C5—C6—C7	109.9 (6)	C37—C36—H36	106.7
C5—C6—C8	113.1 (5)	C38—C36—H36	106.7
C7—C6—C8	110.5 (6)	C36—C37—H37A	109.5
C5—C6—H6A	107.7	C36—C37—H37B	109.5
C7—C6—H6A	107.7	H37A—C37—H37B	109.5
C8—C6—H6A	107.7	C36—C37—H37C	109.5
C6—C7—H7A	109.5	H37A—C37—H37C	109.5
C6—C7—H7B	109.5	H37B—C37—H37C	109.5
H7A—C7—H7B	109.5	C36—C38—C39	115.1 (6)
C6—C7—H7C	109.5	C36—C38—H38A	108.5
H7A—C7—H7C	109.5	C39—C38—H38A	108.5
H7B—C7—H7C	109.5	C36—C38—H38B	108.5
C9—C8—C6	117.9 (6)	C39—C38—H38B	108.5
C9—C8—H8A	107.8	H38A—C38—H38B	107.5
C6—C8—H8A	107.8	C38—C39—C40	118.3 (6)
C9—C8—H8B	107.8	C38—C39—H39A	107.7
C6—C8—H8B	107.8	C40—C39—H39A	107.7
H8A—C8—H8B	107.2	C38—C39—H39B	107.7
C8—C9—C10	118.0 (5)	C40—C39—H39B	107.7
C8—C9—H9A	107.8	H39A—C39—H39B	107.1
C10—C9—H9A	107.8	C41—C40—C39	112.2 (6)
C8—C9—H9B	107.8	C41—C40—C43	111.7 (5)
C10—C9—H9B	107.8	C39—C40—C43	112.8 (5)
H9A—C9—H9B	107.1	C41—C40—H40	106.6
C11—C10—C9	111.1 (5)	C39—C40—H40	106.6
C11—C10—C13	110.9 (5)	C43—C40—H40	106.6
C9—C10—C13	113.9 (5)	C42—C41—C40	110.3 (5)
C11—C10—H10	106.8	C42—C41—H41A	109.6
C9—C10—H10	106.8	C40—C41—H41A	109.6
C13—C10—H10	106.8	C42—C41—H41B	109.6
C12—C11—C10	110.7 (5)	C40—C41—H41B	109.6
C12—C11—H11A	109.5	H41A—C41—H41B	108.1
C10—C11—H11A	109.5	C35—C42—O5	60.4 (4)
C12—C11—H11B	109.5	C35—C42—C32	109.8 (5)
C10—C11—H11B	109.5	O5—C42—C32	111.6 (5)
H11A—C11—H11B	108.1	C35—C42—C41	125.4 (5)
O1—C12—C5	59.9 (4)	O5—C42—C41	116.9 (5)
O1—C12—C11	116.4 (5)	C32—C42—C41	118.9 (6)
C5—C12—C11	125.5 (5)	O6—C43—C45	105.1 (5)
O1—C12—C2	112.1 (5)	O6—C43—C44	108.8 (5)
C5—C12—C2	110.1 (5)	C45—C43—C44	111.3 (5)
C11—C12—C2	118.7 (6)	O6—C43—C40	108.5 (5)
O2—C13—C14	104.8 (5)	C45—C43—C40	112.9 (5)
O2—C13—C15	109.2 (5)	C44—C43—C40	110.0 (5)
C14—C13—C15	110.0 (5)	C43—C44—H44A	109.5
O2—C13—C10	109.1 (5)	C43—C44—H44B	109.5
C14—C13—C10	112.7 (5)	H44A—C44—H44B	109.5
C15—C13—C10	110.8 (5)	C43—C44—H44C	109.5
C13—C14—H14A	109.5	H44A—C44—H44C	109.5
C13—C14—H14B	109.5	H44B—C44—H44C	109.5
H14A—C14—H14B	109.5	C43—C45—H45A	109.5
C13—C14—H14C	109.5	C43—C45—H45B	109.5
H14A—C14—H14C	109.5	H45A—C45—H45B	109.5
H14B—C14—H14C	109.5	C43—C45—H45C	109.5
C13—C15—H15A	109.5	H45A—C45—H45C	109.5
C13—C15—H15B	109.5	H45B—C45—H45C	109.5
H15A—C15—H15B	109.5	C47—C46—H46A	109.5
C13—C15—H15C	109.5	C47—C46—H46B	109.5
H15A—C15—H15C	109.5	H46A—C46—H46B	109.5
H15B—C15—H15C	109.5	C47—C46—H46C	109.5
C17—C16—H16A	109.5	H46A—C46—H46C	109.5
C17—C16—H16B	109.5	H46B—C46—H46C	109.5
H16A—C16—H16B	109.5	C46—C47—C57	115.1 (6)
C17—C16—H16C	109.5	C46—C47—C48	112.5 (5)
H16A—C16—H16C	109.5	C57—C47—C48	102.3 (5)
H16B—C16—H16C	109.5	C46—C47—H47	108.9
C16—C17—C27	114.3 (5)	C57—C47—H47	108.9
C16—C17—C18	114.2 (5)	C48—C47—H47	108.9
C27—C17—C18	102.9 (5)	C49—C48—C47	107.1 (5)
C16—C17—H17	108.4	C49—C48—H48A	110.3
C27—C17—H17	108.4	C47—C48—H48A	110.3
C18—C17—H17	108.4	C49—C48—H48B	110.3
C17—C18—C19	104.8 (5)	C47—C48—H48B	110.3
C17—C18—H18A	110.8	H48A—C48—H48B	108.6
C19—C18—H18A	110.8	C50—C49—C48	103.8 (5)
C17—C18—H18B	110.8	C50—C49—H49A	111.0
C19—C18—H18B	110.8	C48—C49—H49A	111.0
H18A—C18—H18B	108.9	C50—C49—H49B	111.0
C20—C19—C18	105.2 (5)	C48—C49—H49B	111.0
C20—C19—H19A	110.7	H49A—C49—H49B	109.0
C18—C19—H19A	110.7	C57—C50—O7	60.3 (4)
C20—C19—H19B	110.7	C57—C50—C49	110.6 (5)
C18—C19—H19B	110.7	O7—C50—C49	111.0 (5)
H19A—C19—H19B	108.8	C57—C50—C51	127.8 (5)
O3—C20—C27	60.2 (4)	O7—C50—C51	118.2 (5)
O3—C20—C19	108.9 (5)	C49—C50—C51	116.2 (6)
C27—C20—C19	107.9 (5)	C50—C51—C53	115.3 (5)
O3—C20—C21	117.3 (5)	C50—C51—C52	110.2 (5)
C27—C20—C21	127.2 (5)	C53—C51—C52	110.2 (5)
C19—C20—C21	120.0 (5)	C50—C51—H51	106.9
C20—C21—C22	109.9 (5)	C53—C51—H51	106.9
C20—C21—C23	114.0 (5)	C52—C51—H51	106.9
C22—C21—C23	110.3 (5)	C51—C52—H52A	109.5
C20—C21—H21	107.4	C51—C52—H52B	109.5
C22—C21—H21	107.4	H52A—C52—H52B	109.5
C23—C21—H21	107.4	C51—C52—H52C	109.5
C21—C22—H22A	109.5	H52A—C52—H52C	109.5
C21—C22—H22B	109.5	H52B—C52—H52C	109.5
H22A—C22—H22B	109.5	C51—C53—C54	116.2 (6)
C21—C22—H22C	109.5	C51—C53—H53A	108.2
H22A—C22—H22C	109.5	C54—C53—H53A	108.2
H22B—C22—H22C	109.5	C51—C53—H53B	108.2
C21—C23—C24	117.4 (5)	C54—C53—H53B	108.2
C21—C23—H23A	107.9	H53A—C53—H53B	107.4
C24—C23—H23A	107.9	C53—C54—C55	117.7 (5)
C21—C23—H23B	107.9	C53—C54—H54A	107.9
C24—C23—H23B	107.9	C55—C54—H54A	107.9
H23A—C23—H23B	107.2	C53—C54—H54B	107.9
C25—C24—C23	116.9 (5)	C55—C54—H54B	107.9
C25—C24—H24A	108.1	H54A—C54—H54B	107.2
C23—C24—H24A	108.1	C58—C55—C56	112.0 (5)
C25—C24—H24B	108.1	C58—C55—C54	112.9 (5)
C23—C24—H24B	108.1	C56—C55—C54	112.0 (5)
H24A—C24—H24B	107.3	C58—C55—H55	106.5
C24—C25—C26	111.4 (5)	C56—C55—H55	106.5
C24—C25—C28	114.8 (5)	C54—C55—H55	106.5
C26—C25—C28	111.2 (5)	C57—C56—C55	109.2 (5)
C24—C25—H25	106.3	C57—C56—H56A	109.8
C26—C25—H25	106.3	C55—C56—H56A	109.8
C28—C25—H25	106.3	C57—C56—H56B	109.8
C27—C26—C25	110.6 (5)	C55—C56—H56B	109.8
C27—C26—H26A	109.5	H56A—C56—H56B	108.3
C25—C26—H26A	109.5	C50—C57—O7	60.6 (4)
C27—C26—H26B	109.5	C50—C57—C56	125.7 (5)
C25—C26—H26B	109.5	O7—C57—C56	118.8 (5)
H26A—C26—H26B	108.1	C50—C57—C47	110.6 (5)
C20—C27—O3	59.7 (4)	O7—C57—C47	112.0 (4)
C20—C27—C26	125.0 (5)	C56—C57—C47	116.8 (5)
O3—C27—C26	117.4 (5)	O8—C58—C60	106.2 (6)
C20—C27—C17	109.8 (5)	O8—C58—C59	109.7 (5)
O3—C27—C17	112.5 (4)	C60—C58—C59	111.1 (6)
C26—C27—C17	118.6 (5)	O8—C58—C55	105.9 (5)
O4—C28—C29	106.9 (5)	C60—C58—C55	113.2 (5)
O4—C28—C30	110.9 (6)	C59—C58—C55	110.5 (6)
C29—C28—C30	110.1 (6)	C58—C59—H59A	109.5
O4—C28—C25	106.9 (5)	C58—C59—H59B	109.5
C29—C28—C25	111.7 (5)	H59A—C59—H59B	109.5
C30—C28—C25	110.3 (5)	C58—C59—H59C	109.5
C28—C29—H29A	109.5	H59A—C59—H59C	109.5
C28—C29—H29B	109.5	H59B—C59—H59C	109.5
H29A—C29—H29B	109.5	C58—C60—H60A	109.5
C28—C29—H29C	109.5	C58—C60—H60B	109.5
H29A—C29—H29C	109.5	H60A—C60—H60B	109.5
H29B—C29—H29C	109.5	C58—C60—H60C	109.5
C28—C30—H30A	109.5	H60A—C60—H60C	109.5
C28—C30—H30B	109.5	H60B—C60—H60C	109.5
			
C1—C2—C3—C4	155.7 (6)	C42—C32—C33—C34	26.9 (6)
C12—C2—C3—C4	30.0 (7)	C31—C32—C33—C34	151.1 (6)
C2—C3—C4—C5	−30.1 (7)	C32—C33—C34—C35	−26.4 (6)
C12—O1—C5—C4	98.5 (6)	C42—O5—C35—C36	−120.5 (6)
C12—O1—C5—C6	−119.3 (6)	C42—O5—C35—C34	101.2 (6)
C3—C4—C5—O1	−45.1 (7)	C33—C34—C35—C42	16.1 (6)
C3—C4—C5—C12	18.2 (7)	C33—C34—C35—O5	−48.8 (6)
C3—C4—C5—C6	173.2 (6)	C33—C34—C35—C36	172.9 (5)
O1—C5—C6—C7	−62.2 (7)	C42—C35—C36—C37	−131.6 (6)
C12—C5—C6—C7	−134.3 (6)	O5—C35—C36—C37	−58.9 (7)
C4—C5—C6—C7	76.5 (7)	C34—C35—C36—C37	76.5 (7)
O1—C5—C6—C8	61.8 (8)	C42—C35—C36—C38	−5.2 (9)
C12—C5—C6—C8	−10.2 (9)	O5—C35—C36—C38	67.5 (7)
C4—C5—C6—C8	−159.5 (6)	C34—C35—C36—C38	−157.1 (6)
C5—C6—C8—C9	67.6 (8)	C35—C36—C38—C39	64.3 (8)
C7—C6—C8—C9	−168.7 (6)	C37—C36—C38—C39	−169.5 (6)
C6—C8—C9—C10	−62.8 (8)	C36—C38—C39—C40	−68.5 (8)
C8—C9—C10—C11	−21.9 (8)	C38—C39—C40—C41	−12.4 (9)
C8—C9—C10—C13	−148.0 (6)	C38—C39—C40—C43	−139.6 (6)
C9—C10—C11—C12	81.4 (6)	C39—C40—C41—C42	76.7 (7)
C13—C10—C11—C12	−150.9 (5)	C43—C40—C41—C42	−155.5 (5)
C5—O1—C12—C11	117.5 (6)	C36—C35—C42—O5	103.1 (7)
C5—O1—C12—C2	−101.1 (6)	C34—C35—C42—O5	−103.3 (5)
C4—C5—C12—O1	−103.7 (5)	O5—C35—C42—C32	104.3 (5)
C6—C5—C12—O1	104.0 (7)	C36—C35—C42—C32	−152.7 (6)
O1—C5—C12—C11	−102.6 (6)	C34—C35—C42—C32	1.0 (7)
C4—C5—C12—C11	153.7 (6)	O5—C35—C42—C41	−103.6 (6)
C6—C5—C12—C11	1.4 (10)	C36—C35—C42—C41	−0.5 (10)
O1—C5—C12—C2	104.6 (5)	C34—C35—C42—C41	153.2 (6)
C4—C5—C12—C2	0.9 (7)	C35—O5—C42—C32	−101.2 (6)
C6—C5—C12—C2	−151.4 (6)	C35—O5—C42—C41	117.3 (6)
C10—C11—C12—O1	−126.5 (6)	C31—C32—C42—C35	−139.7 (6)
C10—C11—C12—C5	−56.0 (8)	C33—C32—C42—C35	−17.3 (6)
C10—C11—C12—C2	94.7 (6)	C31—C32—C42—O5	−74.7 (7)
C1—C2—C12—O1	−80.1 (7)	C33—C32—C42—O5	47.8 (6)
C3—C2—C12—O1	45.3 (7)	C31—C32—C42—C41	66.0 (7)
C1—C2—C12—C5	−144.8 (5)	C33—C32—C42—C41	−171.6 (5)
C3—C2—C12—C5	−19.4 (7)	C40—C41—C42—C35	−57.2 (8)
C1—C2—C12—C11	60.3 (7)	C40—C41—C42—O5	−128.6 (6)
C3—C2—C12—C11	−174.3 (5)	C40—C41—C42—C32	92.7 (7)
C11—C10—C13—O2	57.1 (6)	C41—C40—C43—O6	53.8 (7)
C9—C10—C13—O2	−176.7 (5)	C39—C40—C43—O6	−178.8 (6)
C11—C10—C13—C14	−58.9 (7)	C41—C40—C43—C45	−62.3 (7)
C9—C10—C13—C14	67.3 (7)	C39—C40—C43—C45	65.1 (7)
C11—C10—C13—C15	177.3 (5)	C41—C40—C43—C44	172.7 (5)
C9—C10—C13—C15	−56.5 (7)	C39—C40—C43—C44	−59.9 (7)
C16—C17—C18—C19	154.2 (5)	C46—C47—C48—C49	147.0 (6)
C27—C17—C18—C19	29.7 (6)	C57—C47—C48—C49	22.9 (6)
C17—C18—C19—C20	−28.6 (6)	C47—C48—C49—C50	−22.5 (6)
C27—O3—C20—C19	100.3 (5)	C57—O7—C50—C49	102.4 (6)
C27—O3—C20—C21	−119.2 (6)	C57—O7—C50—C51	−119.7 (6)
C18—C19—C20—O3	−47.8 (6)	C48—C49—C50—C57	13.4 (6)
C18—C19—C20—C27	16.0 (6)	C48—C49—C50—O7	−51.6 (7)
C18—C19—C20—C21	172.9 (5)	C48—C49—C50—C51	169.6 (5)
O3—C20—C21—C22	−62.3 (7)	C57—C50—C51—C53	−8.4 (9)
C27—C20—C21—C22	−134.2 (6)	O7—C50—C51—C53	64.4 (7)
C19—C20—C21—C22	73.7 (7)	C49—C50—C51—C53	−159.8 (5)
O3—C20—C21—C23	62.1 (7)	C57—C50—C51—C52	−133.9 (6)
C27—C20—C21—C23	−9.8 (8)	O7—C50—C51—C52	−61.1 (7)
C19—C20—C21—C23	−161.8 (5)	C49—C50—C51—C52	74.7 (7)
C20—C21—C23—C24	67.3 (7)	C50—C51—C53—C54	65.9 (7)
C22—C21—C23—C24	−168.5 (5)	C52—C51—C53—C54	−168.7 (5)
C21—C23—C24—C25	−60.4 (8)	C51—C53—C54—C55	−60.1 (8)
C23—C24—C25—C26	−25.0 (7)	C53—C54—C55—C58	−151.6 (6)
C23—C24—C25—C28	−152.5 (6)	C53—C54—C55—C56	−24.1 (8)
C24—C25—C26—C27	84.1 (6)	C58—C55—C56—C57	−149.6 (5)
C28—C25—C26—C27	−146.5 (5)	C54—C55—C56—C57	82.4 (6)
C19—C20—C27—O3	−102.0 (5)	C49—C50—C57—O7	−103.1 (5)
C21—C20—C27—O3	103.3 (6)	C51—C50—C57—O7	104.2 (7)
O3—C20—C27—C26	−103.9 (6)	O7—C50—C57—C56	−106.0 (6)
C19—C20—C27—C26	154.1 (5)	C49—C50—C57—C56	150.9 (5)
C21—C20—C27—C26	−0.7 (9)	C51—C50—C57—C56	−1.8 (10)
O3—C20—C27—C17	105.0 (5)	O7—C50—C57—C47	104.4 (5)
C19—C20—C27—C17	3.0 (6)	C49—C50—C57—C47	1.2 (7)
C21—C20—C27—C17	−151.7 (6)	C51—C50—C57—C47	−151.4 (6)
C20—O3—C27—C26	116.5 (6)	C50—O7—C57—C56	117.0 (6)
C20—O3—C27—C17	−100.6 (6)	C50—O7—C57—C47	−102.0 (6)
C25—C26—C27—C20	−55.0 (7)	C55—C56—C57—C50	−53.7 (8)
C25—C26—C27—O3	−125.6 (5)	C55—C56—C57—O7	−126.6 (5)
C25—C26—C27—C17	93.8 (6)	C55—C56—C57—C47	94.3 (6)
C16—C17—C27—C20	−145.1 (5)	C46—C47—C57—C50	−137.3 (6)
C18—C17—C27—C20	−20.7 (6)	C48—C47—C57—C50	−15.0 (6)
C16—C17—C27—O3	−80.7 (6)	C46—C47—C57—O7	−71.7 (7)
C18—C17—C27—O3	43.8 (6)	C48—C47—C57—O7	50.6 (6)
C16—C17—C27—C26	61.7 (7)	C46—C47—C57—C56	70.1 (7)
C18—C17—C27—C26	−173.8 (5)	C48—C47—C57—C56	−167.6 (5)
C24—C25—C28—O4	−176.1 (5)	C56—C55—C58—O8	52.7 (7)
C26—C25—C28—O4	56.3 (6)	C54—C55—C58—O8	−179.8 (5)
C24—C25—C28—C29	67.4 (7)	C56—C55—C58—C60	−63.3 (7)
C26—C25—C28—C29	−60.2 (7)	C54—C55—C58—C60	64.2 (7)
C24—C25—C28—C30	−55.5 (7)	C56—C55—C58—C59	171.4 (5)
C26—C25—C28—C30	176.9 (5)	C54—C55—C58—C59	−61.1 (7)

### Hydrogen-bond geometry (Å, º)

**Table:**

*D*—H···*A*	*D*—H	H···*A*	*D*···*A*	*D*—H···*A*
O2—H2···O1^i^	0.84	1.99	2.804 (6)	162
O4—H4···O5^ii^	0.84	2.00	2.792 (7)	157
O6—H6···O3	0.84	1.99	2.821 (7)	171
O8—H8···O7^iii^	0.84	2.00	2.795 (7)	158

Symmetry codes: (i) −*x*, *y*+1/2, −*z*+1; (ii) *x*, *y*−1, *z*; (iii) −*x*+2, *y*+1/2, −*z*+2.
